# Book Review

**DOI:** 10.1120/jacmp.v4i1.2546

**Published:** 2003-01-01

## Abstract

**Intravascular Brachytherapy/Fluoroscopically Guided Interventions.** Medical Physics Monograph No. 28. Stephen Balter, Rosanna C. Chan, and Thomas B. Schope, Jr., editors. American Association of Physicists in Medicine Proceedings of the 2002 Summer School, McGill University, Montreal, Quebec, Canada, July 18–21, 2002. 915 pp. ISBN: 1‐930524‐10‐2.

PACS number(s): 87.53.Jw, 87.59.Ci

The editors have compiled a lengthy monograph of the proceedings of a unique American Association of Physicists in Medicine (AAPM) summer school that covered both therapy and diagnostic topics in terms of each field and where there are overlapping issues. This has led to duplication in writing effort and to a great redundancy among the texts from the various authors. That may seem to be the bad news. To be sure, this is not a text to be read linearly. Rather, the fact that many chapters repeat information of others means that the reader who is looking for information or help regarding a particular topic will also get the germane background. That the background material is presented in detail elsewhere in the monograph could be seen as a plus. Thus, the scope of the monograph is comprehensive. This may prove to be a useful volume for educators.

The editors provide a roadmap in their Introduction that closely reflects the chapters to follow. Their observation is that the monograph, by covering interventional fluoroscopy and vascular brachytherapy together, will help medical physicists in each discipline to better understand the topics, issues, technologies, challenges, rationales, and similarities of the other. This is particularly appropriate since the meeting ground is in the same interventional suite. Much practical information is provided regarding the establishment and maintenance (and assurance) of programs, radiation safety and regulatory issues, staffing, and the sociology of involved professionals. This monograph then may prove to be of great use to practicing clinical medical physicists.

The editors have included some historical and theoretical material. The unresolved and non‐completed work is identified critically. The exposition of dosimetry and standard calibration of intravascular brachytherapy sources is detailed and complete. The description of the state of the art in dose estimation in both brachytherapy and in fluoroscopy indicates work left to be done. This material should be of use to researchers.

In the section on Joint Sessions, the first chapter on radiation biology provides a quick and clear review of general radiation biology. The chapter continues on topic about vascular radiation response, including a review of the results of clinical trials supporting vascular brachytherapy. Presented in subsequent chapters are: quantitative angiography to improve interventional accuracy; (a terse) overview of vascular brachytherapy and unresolved issues regarding restenosis; primers on clinical interventional cardiology and interventional radiology (both including useful schematic and radiographic anatomy for the nonphysician); an overview of in‐suite staff radiation safety and concerns and dose management remedies; and federal and state regulatory issues and perspectives. In a sense, the content of these first 261 pages lays out the problems and the terrain of the following 654 pages. The reader will have to reach beyond the first third of the monograph to find the clinically and operationally useful and specialized material.

The next two sections are on the Intravascular Brachytherapy and Fluoroscopically Guided Intervention sessions of the summer school. In the former section, the first chapter covers reactor based production of radioisotopes via neutron activation. The second chapter surveys sources for vascular irradiation: appropriate radionuclides for brachytherapies (in both encapsulated and liquid forms), microsource x‐ray devices, gated and conventional linear accelerator x‐ray sources, and radioactive and activated stents. Realized commercial brachytherapy systems are described in light of the lack of a “truly suitable isotope” for vascular brachytherapy. The third through fifth chapters address dosimetry and calibration of the sources that are (or would/might be) in use for intravascular irradiation. The third chapter covers the TG43/TG60 dosimetry systematics as defined in liquid water and practical measurements of characteristic dose distributions around clinical brachytherapy sources. The chapter serves as a guide for the experimentalist, covering issues of near‐field dosimetry using finite‐size dosimeters (often nearly the same size as sources), the variety of passive and active dosimeters, phantom materials and water equivalency, and procedures for the conversion of measured detector response into reported point and distribution dose. The concise fourth chapter, “Monte Carlo modeling for intravascular brachytherapy sources,” is authoritative, covering pertinent literature, existing models, and uses of the computer codes that implement them, the import of the selection of cross‐section data, and point‐kernel methods in homogeneous phantoms. Standard calibration of available brachytherapy sources is covered in detail in the fifth chapter, including analysis of well chamber response to clinical vascular brachytherapy source trains and a guide for the clinical physicist. The chapter on “Operational IVBT programs” describes commercially available intravascular brachytherapy (IVBT) systems and methods to cover the IVBT treatment zone, and previews the later chapter on IVBT program quality management. General personnel radiation safety and specific emergency procedures for commercially available IVBT systems and devices are covered in the seventh chapter. The state of the art in treatment planning for intravascular brachytherapy is reviewed in the eighth chapter. Issues of actual versus idealized source placement within vessels, dose prescription, and dose calculation methods are addressed and research futures are identified. Perhaps the most useful chapters for the practicing clinical medical physicist are the chapters on quality management and professional roles in vascular brachytherapy. The brevity of these chapters, particularly the chapter on quality management, arises from the authors' clarity. The many details and issues of in‐suite quality management of vascular brachytherapy procedures are outlined. In the chapter on professional roles, the history, regulation and standards, and the proposed delineation of roles and duties are detailed. To the casual reader, this material might seem superfluous; however, the milieu of vascular brachytherapy is in the cardiac catheterization suite where nurses, cardiologists, therapy (and possibly diagnostic) medical physicists, and radiation oncologists form a team that of necessity must be efficient and clearly defined.

The third section of the monograph addresses Fluoroscopically Guided Intervention and follows a somewhat similar exposition as given in the Intravascular Brachytherapy section. There are operational, physical, and technological descriptions of equipment, its uses, and implementation in a fluoro suite, including facilities planning. A chapter discusses the issues specific to equipping a pediatric interventional radiology facility, Picture Archive and Communication Systems, image display, and compression are covered in two chapters. Digital Imaging and Communications in Medicine conformance is discussed with attention to image presentation and calibration (display and print form). The chapter on skin dose estimation provides detail on standards and regulation as well as direct (measured at point of interest) and indirect (inferred) dose measurement techniques, and real‐time dose mapping, all toward timely management of patient dose. This is important when new procedures involve extended exposure to both patient and personnel. The chapter on “Acceptance testing and performance monitoring of interventional radiology systems” provides a comprehensive and authoritative derivation of what a clinical physicist does in acceptance and routine quality control testing, summarizing several AAPM reports in the process. The final two chapters of the monograph relate to accreditation of facilities and programs and to credentialing and training of fluoroscopists. In these, the perspectives the FDA and of external review organizations (i.e., American College of Radiology and the Joint Commission for the Accreditation of Healthcare Organizations) are presented in light of the stances of professional bodies (e.g., the conference of Radiation Control Program Directors, American College of Cardiology). Training, its documentation, and the establishment and management of a credentialing program are addressed.

In summary, this lengthy monograph provides much useful, if often reiterated, material. It may be useful as an educational text. It would be a good reference for a medical physicist working in either vascular brachytherapy or interventional radiology, and would be a good point of departure for one establishing such programs. If the advent of new technologies supplant those addressed in this volume, then its content may be most useful to ensure the correct performance of low frequency procedures. For example, if vascular brachytherapy is principally replaced by pharmaceutical approaches, this volume might be invaluable to the physicist who needs to assist a cardiologist and a radiation oncologist, nursing and catheterization laboratory staff in properly performing a brachytherapy procedure that had become a prior yet infrequently necessary art.

Reviewed by Robert E. Wallace, Ph.D., Professor

UCLA Radiation Oncology

MP200/B265 MC:695124

Los Angeles, CA 90095‐6951

